# The Best Anticoagulation Strategy for Cirrhotic Patients who Underwent Splenectomy: A Network Meta-Analysis

**DOI:** 10.1155/2017/9216172

**Published:** 2017-06-06

**Authors:** Cheng Gong, Xian Qin, Jian Yang, Tao Guo

**Affiliations:** ^1^Department of General Surgery, Zhongnan Hospital of Wuhan University, Wuhan 430071, China; ^2^School of Nursing, Huanggang Polytechnic College, Huanggang, 438002, China

## Abstract

**Objective:**

To determine the best anticoagulation strategy for the patients who underwent splenectomy with cirrhosis through network meta-analysis.

**Methods:**

We conducted a systematic review of the literature in PubMed, Embase, and the Cochrane Library database. We extracted data on incidence of Portal vein system thrombosis (PVST) from studies that compared various anticoagulation strategies for use with patients who underwent splenectomy with cirrhosis. Network meta-analysis was conducted in ADDIS by evaluating the different incidence of PVST. Consistency and inconsistency models were developed to identify differences among the therapeutic strategies. Cumulative probability was utilized to rank the strategies under examination. Results. A total of 11 studies containing 1153 patients were included in the network meta-analysis. The results revealed that the application of Antithrombin III was the best anticoagulation option for patients who underwent splenectomy with cirrhosis (P = 0.59). The data of consistency and inconsistency models exhibited basically consistent and showed good convergence.

**Conclusions:**

Application of Antithrombin III seemed to be the best anticoagulation strategy for cirrhotic patients who underwent splenectomy and should be considered a first-choice clinical reference.

## 1. Introduction

Portal vein system thrombosis (PVST) refers to the blood clots in portal, splenic, superior mesenteric veins or/and intrahepatic portal vein branches, as they form an interactive vascular system without valves [1]. PVST is a life-threatening vascular disease characterized by the development of thrombosis [2, 3]. PVST may lead to liver damage, variceal bleeding with portal hypertension, or ischemic intestinal necrosis. Furthermore, PVST can contribute to more difficult future liver transplantation [4–7]. For patients with liver cirrhosis, PVST is not a rare complication. In addition, obstruction of the portal vein and its tributaries is capable of leading to serious adverse short- and/or long-term events in the affected patients with cirrhosis.

Splenectomy is a therapeutic operation to thrombocytopenia and hypersplenism in patients with cirrhosis. It has been demonstrated to improve the liver function and play a role in the surgical strategy for hepatocellular carcinoma by alleviating thrombocytopenia in cirrhotic patients [8, 9]. Although splenectomy is growing in importance for cirrhotic patients, the indications for splenectomy remain controversial because splenectomy is associated with postoperative complications and this surgical intervention with a conventional laparotomy carries a risk of postoperative liver failure in cirrhotic patients presenting with substantial liver damage [10, 11]. It has been described about PVST following splenectomy, either laparoscopic or laparotomic [12–14]. The complication, is potentially lethal, resulting in ischemic intestinal necrosis or variceal bleeding with portal hypertension [4, 15, 16]. So the use of anticoagulant drugs in perioperative period of splenectomy has become a hot topic of clinical researches.

For the record, the occurrence of bleeding in cirrhotic patients was primarily due to the severity of portal pressure, endothelial dysfunction and bacterial infections, but not the disturbed hemostasis [17]. Accordingly, the prophylactic application of anticoagulation might be theoretically feasible for patients subsequent to splenectomy, including cirrhotic patients who were demonstrated to had high risks of developing PVST after splenectomy [18]. For now, application of various anticoagulation pharmacologic prophylaxis strategies (including warfarin, heparin, aspirin and so on) in perioperative period of splenectomy were proven to be safe and effective for cirrhotic patients. However, the best anticoagulation strategy remains unclear. Therefore, in this study, we performed a network meta-analysis to determine the best anticoagulation strategy for patients who underwent splenectomy with cirrhosis and aimed to provide a clinical reference.

## 2. Methods

### 2.1. Data Sources and Search Strategy

This review was conducted using a predefined protocol and was in accordance with PRISMA and MOOSE guidelines [19, 20]. And the whole methodology was statistically evaluated and discussed in detail. Global databases (Pubmed, EMBASE, and Cochrane Central) were searched until February 1st, 2017. We did not apply any language, publication date, or publication status restrictions. For a more comprehensive and inclusive review, we conducted an initial literature search of respective databases using only a few expressions, such as “splenectomy (or lienectomy and)” and “anticoagulation” Then, we expanded the search terms to include relevant topics to avoid neglecting eligible studies. All abstracts that were available in English and other languages were reviewed. We referred to the full text when necessary to clarify eligibility status.

### 2.2. Study Selection and Eligibility Criteria

The studies included in our meta-analysis satisfied all the following criteria: (1) randomized controlled trial, cohort or observational study with control group; (2) the details of anticoagulation treatment for splenectomy was clearly presented; (3) the cirrhosis was clear diagnosed; (4) the treatment method was the only intervention in the study; (5) outcome information, including the incidence of PVST, was provided; (6) all data for meta-analysis must come from successful operations; (7) English-language titles or abstracts must be searched in globally recognized databases.

The exclusion criteria eliminated studies with the following characteristics: (1) no control group, (2) incomplete raw data for the purposes of this research; (3) without Cirrhosis or mixed diseases; (4) limitation to animals or cells; (5) reviews, study protocols, comments, or case reports; (6) studies unrelated to the prevention of PVST for splenectomy.

### 2.3. Data Extraction and Quality Assessment

Two investigators (Gong C, Yang J) independently reviewed the full manuscripts of eligible studies and entered the extracted information, including publication data (the first author's name, year of publication, and country of the population under examination), treatment methods, and sample size, into an electronic database. Any discrepancies in the extraction of PVST incidence were resolved by the primary investigator (Guo T). Two reviewers (Gong C, Qin X) independently assessed the quality of each study included in the database.

The Grades of Recommendations Assessment, Development and Evaluation (GRADE) system was selected to assess the methodological quality of evidence [21]. Five factors that may reduce the quality of evidence were considered (research limitations, inconsistent findings, uncertain direct evidence, inaccuracy or wide confidence interval, publication bias). At mean time, three factors that may reduce the quality of evidence were also reviewed (effect size, possible confounding factors, dose-effect relationship). Controversial items were discussed with the primary investigator (Guo T) before final consensus was reached.

### 2.4. Statistical Analysis

In this research, we paid close attention to PVST incidence of different interventions. It was necessary to make comparisons across all therapy strategies via a comprehensive network meta-analysis based on Bayesian theorem. This analysis can be considered to be an extension of the traditional pairwise meta-analysis, as it incorporates both direct and indirect information through a common comparator to obtain estimates of relative effects via multiple comparisons.

We evaluated consistency by combining the quantitative estimates from the indirect comparisons, according to the experimental design and primary outcome of the included studies. If there was no evidence of a relevant inconsistency, a consistency model could be used to draw conclusions about the relative effect of the included interventions. A relevant rank probability plot could present the best therapeutic measure. Meanwhile, for analyzing the potential bias of network meta-analysis, node-splitting analysis was also performed to investigate whether a statistically significant inconsistency existed when *P* > 0.05. If node-splitting analysis could not be established, the results of consistency and inconsistency analysis would be presented simultaneously. Convergence was assessed to calculate the potential scale reduction factor (PSRF), the values of which were limited to 1.

The automated software Aggregate Data Drug Information System (ADDIS, version 1.16, GZ Groningen, Netherlands) was used for the network pooled estimation and node-splitting analysis. Explanations for Cochrane Summary of Findings Table of GRADE system was made by software GRADEprofiler (version 3.6, http://www.gradeworkinggroup.org/).

## 3. Results

### 3.1. Study Characteristics and Quality

**Figure 1:**
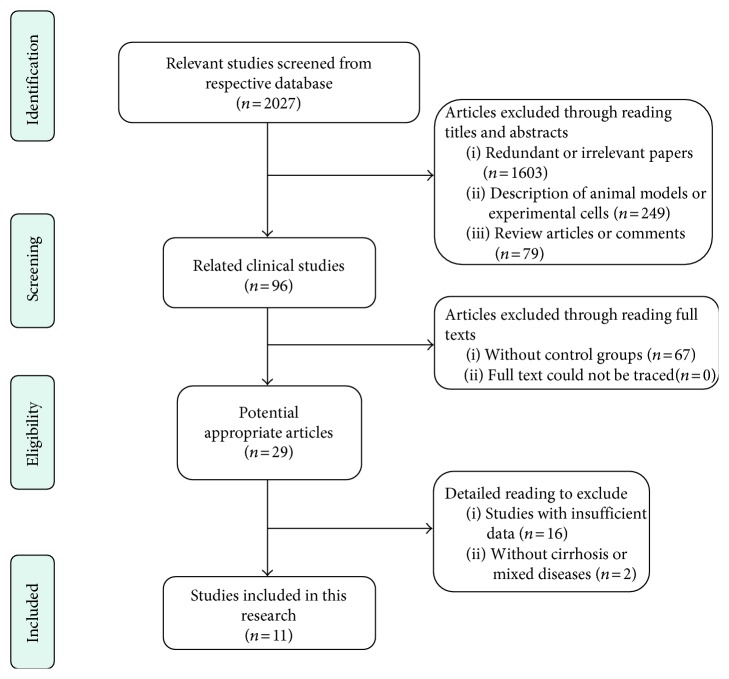
Flow diagram of the process of (and the reasons for) including and excluding studies for this meta-analysis.

**Figure 2:**
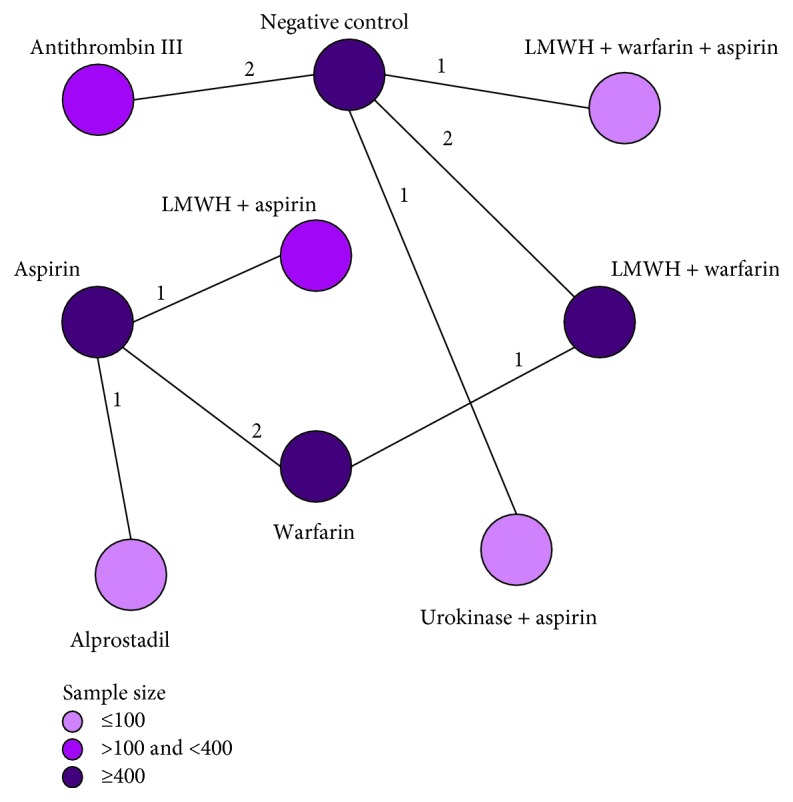
Comparison network of the included studies. Each line connected 2 anticoagulation strategies from original studies. The number on the line refers to the quality of studies comparing each pair of strategies, which were also represented by the width of the lines.

Through the literature search and selection based on the criteria above, we identified 2027 relevant citations, and finally 11 studies (from 2000 to 2016) containing 1153 patients [22–32] were included in this meta-analysis (Figure 1). These 11 Asian studies (Table 1) reported 8 anticoagulation strategies including: Antithrombin III (ATIII), low-molecular-weight heparin (LMWH) plus Warfarin plus Aspirin, LMWH plus Warfarin, Urokinase + Aspirin, Warfarin, Alprostadil, Aspirin, LMWH plus Aspirin. The relationships between each strategy were sorted and presented in Figure 2.

By focusing on the incidence of PVST, based on the relationships between each strategy and GRADE system, we evaluated the quality of the evidence via respective direct comparisons. The evidence showed that three of them revealed high or moderate quality. Meanwhile, the other five comparisons exhibited low or very low quality (Table 2).

### 3.2. Application of Antithrombin III Is the Best Anticoagulation Strategy for Splenectomy

We conducted a network comparison containing the abovementioned 8 anticoagulation strategies by establishing connections between each strategy. After pooled estimation, the network meta-analysis revealed that compared with other anticoagulation strategies, antithrombin III could be the most effective strategy to significantly reduce the incidence of PVST (Rank *P* = 0.59) (Table 3) and was shown to be much better than other methods (Table 4).

### 3.3. Consistency and Convergence Analysis

In this research, node-splitting models were developed to assess inconsistency by testing the difference between the direct and indirect effects. The goal was to determine whether direct and indirect evidence on a specific node (the split node) were in agreement. However, in this research, the node-splitting models could not be established. So we presented all the results of comparisons based on consistency and inconsistency model. Observing the results, we found that the data of consistency and inconsistency model were basically consistent (Table 3). So we deemed that the results were reliable. Moreover, all PSRF values of the different parameters were limited to 1, which demonstrated good convergence and efficiency.

## 4. Discussion

PVST is a common complication after splenectomy and can significantly affect a patient's life expectancy. The occurrence of PVST in cirrhotic patients has been linked to a combination of thrombophilic factors and local inflammation based on the classic Virchow triad [33]. Compared with other surgical interventions, splenectomy is always followed by increased blood viscosity as a result of high platelet and leukocyte counts secondary to absent splenic breakdown. Sequela of splenectomy is increased rigidity of erythrocytes possibly caused by the accumulation of nuclear remnants. Furthermore, in previous studies, the incidence of PVST subsequent to splenectomy differs markedly, ranging from 0.36% to 80% [34, 35] due to lack of typical symptoms, low detection rate or different types of study [36, 37]. Although the prophylaxis of deep vein thrombosis has been relatively well established, the prophylaxis of PVST following splenectomy remains controversial because anticoagulation may induce the anticoagulation-associated bleeding. On the other hand, with the emergence and improvement of different anticoagulant strategies, three meta-analyzes demonstrated prophylactic anticoagulation during perioperative period of splenectomy was safe and effective, even for patients with cirrhosis [38–40]. However, the best or most suitable anticoagulation strategy for patients with cirrhosis has not been addressed.

To the best of our knowledge, this research was the first comprehensive comparison among all reported anticoagulation strategies for cirrhotic patients who underwent splenectomy. According to the incidence of PVST, we performed a network meta-analysis model to determine the best anticoagulation strategy based on the Bayesian theorem. After repeating selection, 11 included articles containing 1153 patients, fulfilled the inclusion criteria. As we can see from the results, among 8 anticoagulation strategies (Figure 2()), it revealed that application of ATIII seemed to be the best anticoagulation strategy for cirrhotic patients who underwent splenectomy (Rank Probability *P* = 0.59) (Table 4). In addition, the node-splitting models could not be established in this research. But the data of consistency and inconsistency model were basically consistent which meant that the result of pooled estimation was reliable but the results still need to be discussed.

As we know, preoperative ATIII activity plays a crucial role in the development of PVST and was found to be an independent predictor of PVST after splenectomy [27]. Cirrhotic patients after splenectomy showed decreased levels of ATIII activity, which are associated with hypercoagulable status, and reduced portal venous flow, resulting from the elimination of increased splenic blood flow. ATIII can prevent PVST without increasing the risks of postoperative hemorrhage after splenectomy and ATIII concentrates can restore the hemostatic balance from a hypercoagulable status to equilibrium [18]. Therefore, the preoperative decrease in ATIII activity and its additional reduction during the early postoperative phase contribute to the development of PVST. Moreover, in liver cirrhosis, hemostatic balance is fragile and easily tips to either a hypo- or hypercoagulable state [41–43]. So application of ATIII was deemed to effect on the formation of PVST factors directly and very suitable for cirrhotic patients.

By contrast, other anticoagulation plan, such as warfarin, it can suppress the synthesis of the specific vitamin K-dependent coagulation factors, II, VII, IX, and X, as well as the two vitamin K-dependent plasma proteins, C and S. It is the predominant oral anticoagulant used for the prevention of recurrent venous thrombus embolism [44, 45]. But warfarin also has disadvantages in preventing postoperative thrombosis, including late usage, slow onset, long duration action duration, and easily caused imbalances in the blood coagulation system [46, 47]. In addition, aspirin is able to prevent thrombosis by inhibiting platelets. However, compared with arterial system, blood flows more slowly in the portal vein system, especially in patients with cirrhosis, making thrombosis less dependent on platelets and anti-platelet therapy less effective in preventing PVST. Coagulation factor Xa is one of the major factor in the procedure of thrombosis [48, 49]. LMWH can suppress factor Xa by combining with ATIII to depress the activation of thrombin and formation of thrombosis so that it can be used for regular anticoagulation [50]. But the activity of ATIII is lower in patients with cirrhosis, which means the application of LMWH may not bring so much effective clinical value. Another anticoagulation plan, alprostadil, we know that it has two major properties. First, alprostadil is a potent antagonist of platelet activation and functions via the platelet prostaglandin receptor by up-regulating adenylate cyclase production of intracellular cyclic adenosine monophosphate. Second, it also inhibits factor VIIa-dependent thrombin formation, impairing thrombus formation [51]. For urokinase, the use of urokinase has been shown to transform plasminogen to plasmin, a strong serine proteinase involved in the degradation of fibrinogen to several fragments. This in turn inhibits platelet aggregation and coagulation, allowing the dose of heparin to be reduced and the associated risk of side effects to be minimized. Thus its application seemed to be more suitable for new thrombosis or thrombolytic therapy. In general terms, compared with ATIII, these above-mentioned drugs, including their combinations, could be used for anticoagulation via indirect or small scale effects. However, for cirrhotic patients, ATIII and its direct systematic effects seemed the most suitable and effective. And our results demonstrated these.

Under the premise of good convergence and consistency, we addressed the best anticoagulation strategy for cirrhotic patients for first time. However, we acknowledge that this research comes with several limitations. First, according to the retrieval principles, we may have overlooked some eligible studies that we were unable to explore. Second, we had included 11 anticoagulation studies, but a larger volume of studies would render better analytical power. Third, although the data of consistency and inconsistency model were basically consistent, the connections among different studies may yield undetected bias or inappropriate comparisons. Furthermore, as discussed above, we only focused on anticoagulation strategies based on the incidence of PVST. Other factors such as the commercial costs and risks of some surgical methods were not included because we deemed some factors to be subordinate and we only included related safety anticoagulation strategies. Last, the quality of evidence of main comparisons was not good enough (5/8 were low or very low) (Table 2). This may bring potential confounding factors to this study. And due to the limited number of included studies, the sensitivity or subgroup analysis according to evidence levels were not able to conduct. Nevertheless, we aim to perform a more comprehensive literature review as more and more researches are reported in the future.

After a network meta-analysis of different anticoagulation strategies for splenectomy, we found that application of ATIII was the best and most suitable for cirrhotic patients. Despite the existence of several limitations, we believed that our final conclusion from this investigation brought some clinical value and should be considered a first-choice clinical reference.

## Figures and Tables

**Table 1:** Characteristics of the included trials.

Author	Year	Country	Study Arms	Surgical procedure	Intervention	Sample Size
Cheng et al. [22]	2015	China	2	Laparoscopic splenectomy and esophagogastric devascularization	LMWH + Aspirin versus Aspirin	139 versus 80
Hongwei et al. [23]	2015	China	2	Splenectomy or with gastroesophageal devascularization	LMWH + Warfarin versus Control	90 versus 46
Jiang et al. [24]	2016	China	2	Laparoscopic splenectomy and azygoportal disconnection	Warfarin versus Aspirin	34 versus 39
Jiang et al. [25]	2016	China	2	Laparoscopic splenectomy and azygoportal disconnection	Warfarin versus Aspirin	35 versus 40
Kakinoki et al. [26]	2013	Japan	2	Hand-assisted laparoscopic splenectomy	LMWH + Warfarin versus Control	14 versus 14
Kawanaka et al. [27]	2010	Japan	2	Laparoscopic splenectomy	Antithrombin III versus Control	25 versus 25
Kawanaka et al. [28]	2014	Japan	2	Laparoscopic splenectomy	Antithrombin III versus Control	37 versus 16
Lai et al. [29]	2012	China	2	Open splenectomy	LMWH + Warfarin versus Warfarin	148 versus 153
Ma et al. [30]	2008	China	2	Splenectomy with gastroesophageal devascularization	Alprostadil versus Aspirin	40 versus 36
Wu et al. [31]	2015	China	2	Open splenectomy	LMWH + Warfarin + Aspirin versus Control	52 versus 19
Xue et al. [32]	2000	China	2	Open splenectomy	Urokinase + Aspirin versus Control	36 versus 35

**Table 2:** Summary of findings and evidence qualities for the main comparisons.

Outcomes	Comparison		No. of trails	Hazard ratio (95% CI)	Sample size	Quality of the evidence (GRADE)
Incidence of PVST	Control	versus Antithrombin III	2	0.05 (0.01, 0.24)	103	⊕ ⊕ ⊕
		versus LMWH + Warfarin + Aspirin	1	0.16 (0.10, 0.43)	191	⊕
		versus Urokinase + Aspirin	1	0.10 (0.02, 0.48)	71	⊕ ⊕ ⊕⊕
		versus LMWH + Warfarin	2	0.40 (0.15, 1.08)	164	⊕
	LMWH + Warfarin	Warfarin	1	2.64 (1.59, 4.40)	301	⊕⊕
	Warfarin	Aspirin	2	2.87 (1.43, 5.73)	148	⊕
	Aspirin	LMWH + Aspirin	1	2.31 (1.31, 4.08)	219	⊕
		Alprostadil	1	6.33 (1.27, 31.67)	76	⊕ ⊕ ⊕

GRADE Working Group grades of evidence.

High quality (⊕⊕⊕⊕): further research is very unlikely to change our confidence in the estimate of effect.

Moderate quality (⊕⊕⊕): further research is likely to have an important impact on our confidence in the estimate of effect and may change the estimate.

Low quality (⊕⊕): further research is very likely to have an important impact on our confidence in the estimate of effect and is likely to change the estimate.

Very low quality (⊕): we are very uncertain about the estimate.

**Table 3:** The network meta-analysis results for different anticoagulation strategies based on consistency and inconsistency model.

Model	Comparisons [Hazard ratio (95% CI)]								
Consistency	Alprostadil	0.10 (0.00, 6.99)	8.45 (0.77, 128.17)	2.38 (0.06, 145.00)	3.71 (0.16, 103.40)	1.00 (0.03, 31.40)	1.35 (0.02, 114.76)	0.19 (0.00, 25.68)	2.81 (0.15, 62.37)
	9.99 (0.14, 1130.26)	Antithrombin III	93.90 (2.49, 4813.16)	24.40 (3.99, 472.28)	39.22 (0.65, 2745.33)	10.29 (0.88, 259.54)	13.86 (0.93, 390.27)	1.95 (0.09, 74.70)	27.86 (1.21, 1225.95)
	0.12 (0.01, 1.31)	0.01 (0.00, 0.40)	Aspirin	0.29 (0.02, 6.31)	0.44 (0.06, 3.10)	0.12 (0.01, 1.37)	0.15 (0.01, 6.56)	0.02 (0.00, 1.17)	0.32 (0.08, 1.37)
	0.42 (0.01, 16.82)	0.04 (0.00, 0.25)	3.40 (0.16, 59.56)	Control	1.57 (0.03, 45.04)	0.40 (0.07, 1.90)	0.52 (0.06, 4.15)	0.07 (0.01, 0.80)	1.14 (0.09, 13.08)
	0.27 (0.01, 6.27)	0.03 (0.00, 1.54)	2.26 (0.32, 17.81)	0.64 (0.02, 28.71)	LMWH + Aspirin	0.27 (0.01, 6.57)	0.34 (0.01, 25.01)	0.05 (0.00, 4.25)	0.74 (0.06, 8.66)
	1.00 (0.03, 32.11)	0.10 (0.00, 1.14)	8.38 (0.73, 96.72)	2.49 (0.53, 13.85)	3.71 (0.15, 84.20)	LMWH + Warfarin	1.22 (0.10, 18.36)	0.19 (0.01, 3.62)	2.68 (0.38, 19.60)
	0.74 (0.01, 53.43)	0.07 (0.00, 1.07)	6.88 (0.15, 194.88)	1.92 (0.24, 16.24)	2.98 (0.04, 138.71)	0.82 (0.05, 9.98)	LMWH + Warfarin + Aspirin	0.14 (0.01, 3.53)	2.18 (0.08, 49.88)
	5.31 (0.04, 447.86)	0.51 (0.01, 10.55)	48.41 (0.85, 1800.44)	13.46 (1.25, 176.53)	21.07 (0.24, 1149.69)	5.34 (0.28, 106.06)	7.25 (0.28, 191.30)	Urokinase + Aspirin	15.47 (0.43, 443.76)
	0.36 (0.02, 6.50)	0.04 (0.00, 0.83)	3.08 (0.73, 13.31)	0.88 (0.08, 11.67)	1.36 (0.12, 15.82)	0.37 (0.05, 2.63)	0.46 (0.02, 12.69)	0.06 (0.00, 2.33)	Warfarin

Inconsistency	Alprostadil	0.10 (0.00, 7.41)	7.36 (0.72, 105.37)	2.44 (0.05, 132.85)	3.23 (0.17, 77.61)	0.95 (0.03, 33.79)	1.34 (0.01, 119.83)	0.18 (0.00, 19.28)	2.51 (0.15, 52.78)
	9.53 (0.13, 958.69)	Antithrombin III	73.89 (2.45, 3679.52)	22.88 (3.65, 271.11)	33.18 (0.75, 2209.94)	8.93 (0.76, 174.82)	12.20 (0.71, 306.30)	1.98 (0.05, 51.21)	25.30 (1.09, 867.87)
	0.14 (0.01, 1.38)	0.01 (0.00, 0.41)	Aspirin	0.31 (0.02, 6.14)	0.44 (0.06, 3.09)	0.12 (0.01, 1.38)	0.17 (0.00, 6.45)	0.02 (0.00, 1.21)	0.33 (0.07, 1.50)
	0.41 (0.01, 19.75)	0.04 (0.00, 0.27)	3.19 (0.16, 64.38)	Control	1.42 (0.04, 46.67)	0.39 (0.07, 2.11)	0.52 (0.06, 4.62)	0.08 (0.01, 0.85)	1.07 (0.08, 13.98)
	0.31 (0.01, 6.03)	0.03 (0.00, 1.33)	2.27 (0.32, 16.51)	0.70 (0.02, 23.27)	LMWH + Aspirin	0.28 (0.01, 6.36)	0.37 (0.01, 23.66)	0.06 (0.00, 4.16)	0.76 (0.06, 8.86)
	1.05 (0.03, 31.79)	0.11 (0.01, 1.32)	8.08 (0.73, 97.15)	2.59 (0.47, 14.36)	3.53 (0.16, 81.97)	LMWH + Warfarin	1.35 (0.08, 19.12)	0.20 (0.01, 3.98)	2.70 (0.37, 19.49)
	0.75 (0.01, 72.23)	0.08 (0.00, 1.41)	6.01 (0.15, 289.21)	1.90 (0.22, 17.38)	2.68 (0.04, 175.22)	0.74 (0.05, 11.94)	LMWH + Warfarin + Aspirin	0.15 (0.00, 4.10)	1.95 (0.07, 66.30)
	5.47 (0.05, 514.46)	0.50 (0.02, 18.67)	40.04 (0.82, 2003.35)	12.68 (1.18, 184.57)	17.65 (0.24, 1362.70)	5.04 (0.25, 112.25)	6.69 (0.24, 204.67)	Urokinase + Aspirin	13.57 (0.38, 497.85)
	0.40 (0.02, 6.53)	0.04 (0.00, 0.92)	3.01 (0.66, 13.67)	0.94 (0.07, 12.23)	1.32 (0.11, 15.48)	0.37 (0.05, 2.72)	0.51 (0.02, 14.54)	0.07 (0.00, 2.65)	Warfarin

**Table 4:** Results of different ranks for anticoagulation strategies.

Strategy	Probability *P* Palues								
	Rank 9	Rank 8	Rank 7	Rank 6	Rank 5	Rank 4	Rank 3	Rank 2	Rank 1
Alprostadil	0.02	0.06	0.09	0.11	0.12	0.19	0.24	0.09	0.08
Antithrombin III	0	0	0	0.01	0.01	0.02	0.05	0.32	0.59
Aspirin	0.72	0.16	0.06	0.02	0.02	0.01	0.01	0	0
Control	0.09	0.16	0.17	0.28	0.21	0.08	0.02	0	0
LMWH + Aspirin	0.09	0.39	0.19	0.12	0.09	0.05	0.03	0.02	0.01
LMWH + Warfarin	0	0.01	0.03	0.07	0.22	0.35	0.27	0.04	0
LMWH + Warfarin + Aspirin	0.06	0.07	0.08	0.12	0.17	0.2	0.24	0.05	0.01
Urokinase + Aspirin	0.01	0.01	0.01	0.02	0.03	0.05	0.12	0.46	0.3
Warfarin	0.01	0.15	0.36	0.24	0.15	0.06	0.02	0.01	0
